# Cerebellar hypoperfusion in anti-NF155 antibody-positive nodopathy: a retrospective case series using brain perfusion SPECT

**DOI:** 10.3389/fneur.2026.1774074

**Published:** 2026-03-25

**Authors:** Hanna Okada, Yuji Tomizawa, Yasunobu Hoshino, Davide Cossu, Hidenori Ogata, Noriko Isobe, Akifumi Hagiwara, Kazumasa Yokoyama, Taku Hatano, Nobutaka Hattori

**Affiliations:** 1 Department of Neurology, Faculty of Medicine, Juntendo University, Tokyo, Japan; 2 Department of Neurology, Graduate School of Medical Sciences, Kyushu University, Kyushu, Japan; 3 Department of Radiology, Faculty of Medicine, Juntendo University, Tokyo, Japan; 4 Tohsei Center for Neurological Diseases, Shizuoka, Japan; 5 Neuron-Glia Crosstalk Center, Faculty of Medicine, Juntendo University, Tokyo, Japan

**Keywords:** 3D-SSP, brain perfusion SPECT, cerebellar hypoperfusion, chronic inflammatory demyelinating polyradiculoneuropathy, neurofascin-155, sensory ataxia

## Abstract

**Introduction:**

Anti–neurofascin-155 (NF155) antibody-positive nodopathy is a distinct autoimmune neuropathy characterized by sensory ataxia, tremor, and poor response to intravenous immunoglobulin (IVIg). Although classically considered a peripheral disorder, central nervous system (CNS) involvement has been suggested, but functional neuroimaging correlates remain unclear.

**Methods:**

We retrospectively analyzed seven male patients with NF155 antibody-positive nodopathy. Clinical and electrophysiological assessments were performed, and all patients underwent brain perfusion single-photon emission computed tomography (SPECT) using 123I-IMP iodoamphetamine. Imaging data were processed using three-dimensional stereotactic surface projection (3D-SSP) and compared to an age-matched database. Cerebellar hypoperfusion was defined as regional *z*-scores ≤ −2.0.

**Results:**

The mean patient age was 39.0 ± 12.9 years, and mean disease duration was 5.0 ± 3.9 years. Tremor was documented in all patients at some point during the disease course and was predominantly postural or kinetic. Cerebellar hypoperfusion was identified in six patients (86%), involving the cerebellum bilaterally in most cases, with variable degrees of asymmetry across individuals. No correlation was found between hypoperfusion and disease duration or age.

**Conclusion:**

Cerebellar hypoperfusion was frequently observed in this single-center case series of anti-NF155 antibody-positive nodopathy. These findings are descriptive and hypothesis-generating, and compatible with a possible association between cerebellar perfusion abnormalities and tremor. Larger prospective studies are warranted to validate these observations and explore their clinical implications.

## Introduction

Anti-neurofascin-155 (NF155) antibody-positive nodopathy represents a distinct subgroup within the spectrum of autoimmune peripheral neuropathies. Previously classified under chronic inflammatory demyelinating polyradiculoneuropathy (CIDP), this condition has been redefined as an autoimmune nodopathy in the revised 2021 European Academy of Neurology/Peripheral Nerve Society (EAN/PNS) guidelines due to its unique clinical, electrophysiological, immunological, and therapeutic characteristics (1).

NF155 nodopathy is characterized by the presence of IgG4 autoantibodies directed against neurofascin-155, a cell adhesion molecule located at the paranodal axo-glial junctions of myelinated fibers. Clinically, patients frequently present with younger age at onset, male predominance, distal-dominant weakness, sensory ataxia, and notably, a high prevalence of tremor (2–4). In some cases, these motor symptoms prompt further evaluation for potential central involvement. Despite its classification as a peripheral demyelinating neuropathy, mounting evidence suggests that NF155 nodopathy may also involve the central nervous system (CNS). NF155 is expressed not only in Schwann cells but also in oligodendrocytes, particularly in cerebellar white matter (5–7). Furthermore, patient sera have been shown to specifically label cerebellar Purkinje cells on mouse brain sections (3). These findings raise the possibility that cerebellar dysfunction may be associated with tremor in anti-NF155 antibody-positive nodopathy; however, causal inference cannot be made from the present data. Notably, growing pathological and immunological evidence implicating the CNS, functional neuroimaging data in NF155 nodopathy remain scarce, and the significance of possible cerebellar involvement has not been fully elucidated.

In this study, we retrospectively analyzed clinical, electrophysiological, and imaging data from patients with anti-NF155 antibody-positive nodopathy (8). During the study period, all patients were diagnosed with anti-NF155 antibody-positive nodopathy at our institution. All patients exhibited tremor at some point during their disease course and underwent brain perfusion single-photon emission computed tomography (SPECT) as part of the clinical evaluation. The presence, type, and timing of tremor varied across patients, with some presenting tremor early in their disease and others showing it later. SPECT imaging was performed due to the presence of tremor, although the timing of tremor varied significantly among patients. This study investigates whether cerebellar hypoperfusion detected by brain perfusion SPECT is associated with tremor and related motor manifestations in this single-center case series of anti-NF155 antibody–positive nodopathy.

## Methods

### Study design and participants

This retrospective case series was conducted at Juntendo University Hospital and included seven male patients diagnosed with anti-neurofascin-155 (NF155) antibody-positive nodopathy. During the study period, seven patients were diagnosed with anti-NF155 antibody-positive nodopathy at our institution, and all were included in the present analysis. All patients had documented tremor at some point during their disease course and underwent brain perfusion single-photon emission computed tomography (SPECT) as part of the clinical evaluation. All seven patients tested positive for serum IgG4 anti-NF155 antibodies using a standardized cell-based assay. Antibody testing was performed at Kyushu University in six patients and at Dokkyo Medical University in one patient (Patient F), using the same standardized IgG4 subclass-specific assay protocol (2, 3, 9). Inclusion criteria comprised confirmed anti-NF155 IgG4 seropositivity and clinical features consistent with chronic inflammatory demyelinating polyradiculoneuropathy (CIDP) based on the 2021 EAN/PNS criteria (1). Patients were subclassified into typical or distal CIDP phenotypes based on clinical and electrophysiological features (10). Exclusion criteria included coexisting neurological or systemic disorders that could affect motor function or cerebral perfusion.

### Clinical and electrophysiological assessment

All patients underwent a detailed neurological examination, nerve conduction studies (NCS), and cerebrospinal fluid (CSF) analysis. Motor and sensory deficits were systematically documented, including presence and laterality of weakness, ataxia, and tremor. Tremor was categorized as resting, postural, or kinetic by two independent neurologists based on bedside clinical examination. In cases with severe proprioceptive loss, movements were carefully distinguish tremor from pseudoathetosis or other sensory-induced abnormal movements. Disease duration was defined as the interval between symptom onset and the time of brain imaging.

### SPECT imaging protocol

Cerebral perfusion imaging was performed in all patients using SPECT with N-isopropyl-p-[123I] iodoamphetamine. Images were acquired using a dual-head gamma camera (Symbia T series, Siemens Healthcare) equipped with low-energy high-resolution collimators. Early-phase images were obtained 15 min after tracer injection. Voxel-wise statistical analysis of brain perfusion was conducted using 3D-stereotactic surface projection (3D-SSP) (11), which compared each patient’s data to an age-matched normative database. Regions of interest (ROIs) were automatically delineated bilaterally in the cerebellum, thalamus, basal ganglia, and cerebral cortex. Cerebellar hypoperfusion was defined as regional z-scores exceeding 2.0 below the normative mean. All images were acquired using the same scanner and acquisition protocol.

### Data collection and statistical analysis

Demographic and clinical data collected included age at imaging, disease duration, clinical phenotype, tremor characteristics, NCS parameters, CSF protein levels, and treatment response. Quantitative imaging metrics (*z*-scores) were extracted to determine whether regional values exceeded the predefined threshold (*z* ≤ −2.0).

Due to the small sample size, no formal between-patient statistical comparisons were performed.

## Results

A total of seven male patients with anti-NF155 antibody-positive nodopathy were included in the study. The mean age at the time of imaging was 39.0 ± 12.9 years, and the mean disease duration was 5.0 ± 3.9 years. Based on clinical and electrophysiological features, five patients were classified as having typical CIDP and two as distal CIDP.

**Figure 1:**
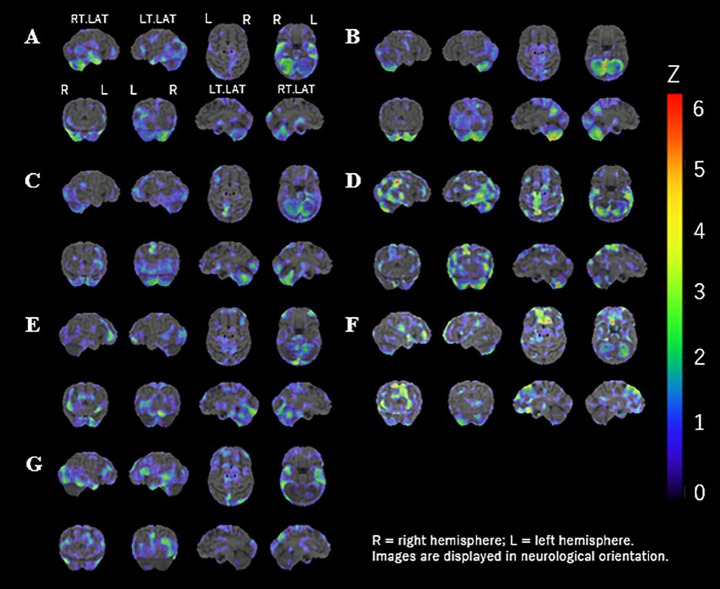
Three-dimensional stereotactic surface projection (3D-SSP) images of brain perfusion in patients with anti-NF155 antibody–positive nodopathy **(A–G)**. Areas of relative hypoperfusion are displayed using a standardized *Z*-score color scale (right; threshold *Z* ≤ −2.0) referenced to an age-matched normative database. Laterality was assessed descriptively based on visual inspection and was not quantitatively analyzed.

Brain perfusion SPECT revealed cerebellar hypoperfusion in six of the seven patients (6/7, 86%), involving the cerebellum bilaterally in most cases, with variable degrees of asymmetry across individuals. However, due to the absence of a disease control group, the specificity of these findings to anti-NF155 nodopathy cannot be definitively established. Cerebellar hypoperfusion was quantified using 3D-SSP z-scores with a threshold of *z* ≤ −2.0. Laterality was assessed descriptively by visual inspection and was not formally quantified. No significant correlation was found between cerebellar hypoperfusion z-scores and patient age (Spearman’s *ρ* = −0.18, *p* = 0.67) or disease duration (*ρ* = −0.24, *p* = 0.58) (Figure 1). Among patients with cerebellar hypoperfusion, two also exhibited additional signs suggestive of CNS involvement, including nystagmus and dysarthria.

Tremor was documented in all seven patients during the disease course. The type and distribution of tremor varied among individuals. Unilateral predominance was noted in three cases, whereas bilateral involvement was observed in the remainder. In one patient, tremor was contralateral to the side of predominant weakness and sensory loss; in the remaining cases, no consistent lateralization of tremor in relation to motor or sensory deficits was identified. Tremor was predominantly postural or kinetic. One patient exhibited a transient unilateral resting tremor. Dopamine transporter imaging was normal in this patient. The patient with minimal cerebellar hypoperfusion also demonstrated bilateral tremor. Individual clinical and imaging characteristics are summarized in Table 1.

**Table 1:** Brain perfusion SPECT findings in patients with anti-NF155 antibody–positive nodopathy.

#	Age	Sex	Onset age	Duration (years)	CIDP subtype	INCAT UE	INCAT LE	Cerebellar feature and oculomotor signs	Abnormal involuntary movement	Deep sensation	Cerebellar hypoperfusion (visual)	Treatment at imaging	CSF protein (mg/dL)
A	42	M	40	2	Typical	4	0	Bilateral decomposition	Postural tremor	Disturbed	Bilateral (R > L)	PSL 7.5 mg	325
B	29	M	24	5	Typical	2	2	Mild left-sided limb ataxia	Bilateral postural tremor	Disturbed	Bilateral (R > L)	PSL 5 mg + cyclosporine 300 mg	920
C	54	M	50	4	Typical	2	1	Left-sided decomposition	Bilateral postural and kinetic tremor	Disturbed	Bilateral (L > R)	PSL6.5 mg	447
D	31	M	28	3	Distal	3	1	Gaze-evoked nystagmus	Bilateral postural tremor	Disturbed	Diffuse bilateral	PSL 5 mg + cyclosporine 150 mg	206
E	32	M	23	9	Typical	1	0	Not present	Bilateral postural and kinetic tremor	Disturbed	Mild bilateral (L ≥ R)	PSL 5 mg	361
F	57	M	44	13	Distal	4	3	Bilateral decomposition of movement	Bilateral postural and kinetic tremor	Disturbed	Mild bilateral (L ≥ R)	Plasmapheresis +PSL5mg	180
G	53	M	51	2	Typical	2	2	Decomposition	Bilateral postural and kinetic tremor, transient unilateral resting tremor	Disturbed	Minimal	PSL9mg + Tacrolimus	125

Three-dimensional stereotactic surface projection (3D-SSP) images are shown for each patient (A–G). Areas of relative hypoperfusion are displayed using a standardized Z-score color scale (threshold *z* ≤ −2.0) referenced to an age-matched normative database. Cerebellar hypoperfusion was observed in the majority of patients, most commonly involving both cerebellar hemispheres. In some individuals, apparent hemispheric predominance was noted on visual inspection; however, the laterality and extent of hypoperfusion varied across patients and were not quantitatively compared. These imaging findings are descriptive and exploratory in nature. Laterality was assessed visually and should be interpreted cautiously. CIDP, chronic inflammatory demyelinating polyneuropathy; INCAT, inflammatory neuropathy cause and treatment score; UE, upper extremity; LE, lower extremity; CSF, cerebrospinal fluid.

## Discussion

This study describes a high frequency of cerebellar hypoperfusion in a clinically selected group of patients with anti-NF155 antibody-positive nodopathy who underwent brain perfusion SPECT, raising the possibility of cerebellar involvement in tremor generation and related motor manifestations (8, 12).

Tremor was the most common motor symptom observed in this cohort. Although tremor in neuropathies is generally attributed to peripheral nerve dysfunction, the present findings support a more complex pathophysiological model that may include cerebellar pathways (3). In addition, in patients with severe sensory neuropathy, sensory ataxia or pseudoathetosis may clinically resemble tremor, and a clear distinction could not be made in a retrospective dataset. Tremor was observed in all patients during the disease course, although its temporal relationship with imaging was variable. In at least one patient, tremor had improved following immunotherapy prior to SPECT acquisition, limiting interpretation of active tremor–perfusion correspondence. The single patient who transiently exhibited resting tremor had normal dopamine transporter imaging and no persistent parkinsonian features, making degenerative parkinsonism unlikely. While cerebellar hypoperfusion was frequently observed, the degree and laterality of hypoperfusion varied across individuals. No consistent clinico-imaging laterality correspondence was identified. These observations should therefore be interpreted in the context of the study’s descriptive and exploratory design. Given the variability in laterality between clinical and imaging findings, further investigation is needed to clarify the role of cerebellar dysfunction in the pathophysiology of tremor in anti-NF155 antibody-positive nodopathy. Larger cohorts and standardized assessments are required to better understand these relationships. Collectively, these clinical and neuroimaging observations are compatible with the hypothesis that cerebellar dysfunction may be associated with tremor in anti-NF155 antibody-positive nodopathy; however, causal inference cannot be made from the present data.

Previous reports have shown that neurofascin-155 is expressed not only in Schwann cells but also in oligodendrocytes, particularly in the cerebellar white matter (7, 12). In addition, experimental studies have demonstrated that sera from anti-NF155-positive patients can label cerebellar Purkinje cells in murine brain tissue, raising the possibility that autoantibody-mediated central damage may occur in parallel with peripheral neuropathy (3, 5). In this context, the regional cerebellar hypoperfusion observed in our cohort may reflect functional alterations within cerebellar circuits implicated in tremor generation (8).

While cerebellar hypoperfusion was common among patients, the relationship between the degree of hypoperfusion and tremor severity remains unclear. The high frequency and distribution of hypoperfusion findings suggest a possible cerebellar contribution to the clinical phenotype. Visual inspection of SPECT images demonstrated heterogeneous patterns of cerebellar involvement, with some patients showing apparent hemispheric predominance and others exhibiting bilateral or diffuse hypoperfusion. Given the small sample size and descriptive design, these observations should be interpreted cautiously.

From a therapeutic standpoint, recognition of possible cerebellar involvement in NF155 nodopathy may have clinical implications. Patients with anti-NF155 antibodies often show poor responses to intravenous immunoglobulin (IVIg) therapy (2, 3). Whether cerebellar hypoperfusion represents a distinct immunopathological subtype or predicts differential treatment response remains unclear. However, follow-up SPECT imaging after immunotherapy was not systematically performed, and therefore changes in cerebellar perfusion over time could not be evaluated in this retrospective series.

Several limitations of this study should be considered. All patients included in this analysis underwent SPECT during the study period; however, the small sample size and single-center design limit the generalizability of the findings. In at least one patient, tremor had improved following immunotherapy prior to imaging. This temporal variability limits interpretation of the relationship between active tremor and perfusion abnormalities. Second, the absence of an age-matched disease control group precludes direct comparison of perfusion patterns. Third, the lack of standardized quantitative tremor rating scales and objective motor assessments may have limited phenotypic resolution. In the context of severe sensory neuropathy, some motor findings may reflect overlapping sensory ataxia and cerebellar contributions, which cannot be fully disentangled in this retrospective analysis. Finally, while SPECT imaging provides functional information, it cannot confirm structural lesions or directly demonstrate antibody-mediated CNS involvement (8). Future prospective studies with larger cohorts, inclusion of appropriate disease controls, objective tremor quantification, and advanced neuroimaging modalities are needed to clarify the clinical relevance of cerebellar involvement in anti-NF155 antibody-positive nodopathy.

## Conclusion

This study highlights a high frequency of cerebellar hypoperfusion in this single-center case series of patients with anti-NF155 antibody-positive nodopathy who presented with prominent tremor and/or ataxia and underwent brain perfusion SPECT. These findings are descriptive and hypothesis-generating and do not establish disease specificity or causality. Further prospective, multicenter studies incorporating appropriate disease controls and quantitative motor assessments are needed to clarify the clinical and pathophysiological significance of cerebellar involvement in this condition.

## Data Availability

Anonymized data that support the findings of this study are available from the corresponding author upon reasonable request.
